# Metabolic and Epigenetics Action Mechanisms of Antiobesity Medicinal Plants and Phytochemicals

**DOI:** 10.1155/2021/9995903

**Published:** 2021-06-09

**Authors:** Bashar Saad, Bilal Ghareeb, Abdalsalam Kmail

**Affiliations:** ^1^Faculties of Medicine and Arts and Sciences, Arab American University, P.O. Box 240, Jenin, State of Palestine; ^2^Qasemi Research Center, Al-Qasemi Academy, P.O. Box 124, 30100 Baqa Al-Gharbia, Israel

## Abstract

Ever-growing research efforts are demonstrating the potential of medicinal plants and their phytochemicals to prevent and manage obesity, either individually or synergistically. Multiple combinations of phytochemicals can result in a synergistic activity that increases their beneficial effects at molecular, cellular, metabolic, and temporal levels, offering advantages over chemically synthesized drug-based treatments. Herbs and their derived compounds have the potential for controlling appetite, inhibiting pancreatic lipase activity, stimulating thermogenesis and lipid metabolism, increasing satiety, promoting lipolysis, regulating adipogenesis, and inducing apoptosis in adipocytes. Furthermore, targeting adipocyte life cycle using various dietary bioactives that affect different stages of adipocyte life cycle represents also an important target in the development of new antiobesity drugs. In this regard, different stages of adipocyte development that are targeted by antiobesity drugs can include preadipocytes, maturing preadipocytes, and mature adipocytes. Various herbal-derived active compounds, such as capsaicin, genistein, apigenin, luteolin, kaempferol, myricetin, quercetin, docosahexaenoic acid, quercetin, resveratrol, and ajoene, affect adipocytes during specific stages of development, resulting in either inhibition of adipogenesis or induction of apoptosis. Although numerous molecular targets that can be used for both treatment and prevention of obesity have been identified, targeted single cellular receptor or pathway has resulted in limited success. In this review, we discuss the state-of-the-art knowledge about antiobesity medicinal plants and their active compounds and their effects on several cellular, molecular, and metabolic pathways simultaneously with multiple phytochemicals through synergistic functioning which might be an appropriate approach to better management of obesity. In addition, epigenetic mechanisms (acetylation, methylation, miRNAs, ubiquitylation, phosphorylation, and chromatin packaging) of phytochemicals and their preventive and therapeutic perspective are explored in this review.

## 1. Introduction

Diet-induced obesity is a main aspect of the modern sedentary lifestyle, dietary habits, and metabolic dysfunctions described globally as the cardiometabolic risk factors syndrome. It is a multidimensional condition ranging from adipocyte hypertrophy to the appearance of metabolic alteration-associated symptoms. This syndrome entails insulin resistance, dyslipidemia, abdominal obesity, and hypertension [1, 2]. Consequently, the global prevalence of diet-induced obesity, so far considered realistically as a global epidemic, is a main health concern and a leading cause of morbidity and mortality on a global scale. It leads to increasing the risk of contracting several medical complications not only the aforementioned conditions but also cancer among other complications [3–5]. In addition, obesity enhances chronic inflammation in adipose tissue besides skeletal muscles, liver, and the vascular system. Consequently, chronic inflammation stimulates the release of proinflammatory cytokines, for example, tumor necrosis factor-*α* (TNF-*α*), monocyte chemoattractant protein-1 (MCP-1), and interleukin-6 (IL-6). The obesity-enhanced immune cell infiltration is intimately linked to insulin resistance (IR) via interference with the insulin signaling pathway in adipose tissue and skeletal muscle. It is also linked to the development of atherosclerosis in vessel walls [6–8].

The caloric imbalance in form of obesity is classically handled by caloric intake restriction, physical exercising, and pharmacological drugs. Surprisingly, the majority (more than 90%) of individuals who lose weight following dieting restore the original weight within 5 years. This has been pointed out in human society worldwide where the use of medicinal plants, pharmaceuticals, and dieting targeting body weight loss is becoming more and more familiar. The plant-based pharmaceutical approach is the safe and efficient promising approach that is exponentially gaining a worldwide agreeable interest [9–14]. Consequently, there is a strong growing body of evidence-based research on the virtues of phytochemicals in prevention as well as treatment of obesity and its related chronic complications and diseases. Compared to treatments based on synthetic drugs, medicinal plants are valuable in providing an increased bioavailability of phytochemicals with beneficial additive and synergistic effects on the level of the whole organism as well as the cellular and molecular levels. Contrary to the primary chemical components of plants (carbohydrates, proteins, lipids, nucleic acid, and chlorophyll) which are found at similar percentages in plants, phytochemicals are groups distinct. Consequently, the herbal pharmacological actions of plants' secondary products and their combinations are taxonomically specific.

**Figure 1:**
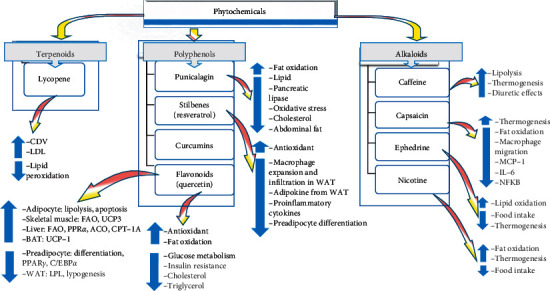
Phytochemicals and their antiobesity action mechanisms. Docosahexaenoic acid (DHA), conjugated linoleic acid (CLA), (–)–hydroxycitric acid (HCA), and (–)–epigallocatechin gallate (EGCG).

The plant secondary metabolites or active compounds have naturally initially evolved throughout millions of years for protective and defensive roles besides the metabolic ones [15–17]. They enable plants to withstand variable environmental insults such as UV radiation, toxicity, and pathogens [18, 19]. The aforementioned virtues of phytochemicals for the plant can be extrapolated (at least partially) on the human health and coping mechanisms through, simply, dietary intake. Our organism reacts evidently to these stress-induced plant compounds [20, 21]. The modern global interest in these safe compounds has characterized, so far, thousands originating from a multitude of plants [22]. They can be classified into the main categories terpenoids, phenolics, and alkaloids with exponentially increasing therapeutic as well as preventive virtues [1–5] Figure 1.

More than 20,000 secondary metabolites phytochemicals are categorized in the group of terpenoids (isoprenoids) which are, therefore, considered as one of the biggest secondary metabolites groups. Many terpenoids are reported for their pharmaceutical applications. For example, artemisinin acting as antimalarial and taxol acting as anticancer drugs are just examples of terpenoids (Figure 1). The mechanism of action of several terpenoids passes through modulation of ligand-dependent transcription factors activities. One example is peroxisome proliferator-activated receptors (PPARs) which are dietary lipid sensors that are responsible for controlling energy homeostasis. It has been reported that continuous consumption of these terpenoids on a daily basis could be helpful in the management of several obesity-related metabolic disorders. Examples of disorders that responded positively to such treatment include hyperlipidemia, type 2 diabetes, and cardiovascular diseases [3, 23–25].

Polyphenols represent the second largest secondary metabolites group with more than 8,000 various polyphenolic substances characterized so far. They share similar chemical structures, with, however, distinctive characteristics, and consequently, polyphenols are categorized into two subgroups, flavonoids and nonflavonoids, for example, tannins [26]. At the cellular and molecular levels, it has been shown that adipocyte viability and preadipocyte proliferation are significantly reduced under the effect of polyphenolic secondary metabolites. In addition, these plant-derived chemicals have an inhibitory effect on adipocyte differentiation, triglyceride accumulation, and inflammation. Polyphenols have a stimulatory effect on lipolysis and fatty acid *β*-oxidation.

Experiments on animals demonstrated that plant-derived polyphenols play an important role in reducing obesity and animals' weight as well as triglycerides through inducing fat use and energy expenditure, in addition to tuning glucose homeostasis. Importantly, experiments accomplished on humans did show inconsistent results concerning the antiobesity effects of polyphenols. The reasons for these results discrepancies can be due to variations among experimental subjects, experimental design, duration, and chemical forms. More investigations taking into consideration these factors (notably using randomized controlled trials) can yield more conclusive results on the pharmaceutical virtues of the herbal-derived polyphenols [3, 23, 24, 27, 28].

It has recently been demonstrated by numerous investigators that dietary polyphenols can exert an important role in the management of obesity and obesity-linked chronic disorders (Figure 1). For instance, green tea catechins, notably epigallocatechin gallate (EGCG), curcumin, and resveratrol, which are worldwide commonly and traditionally used dietary elements, are efficient in treating obesity as well as obesity-linked inflammation. *Hibiscus sabdariffa* (which is notorious for its multiuses worldwide including the orient, where it is used as a fresh and hot beverage) has been the topic of an excellent review by Herranz-López et al. [29] on the preventive antiobesity virtues of the polyphenols highly available in this plant. All the available phenolic compounds extracted from *Hibiscus sabdariffa* were assembled and analyzed for their virtues in a perspective of antiobesity-associated pathologies [27–33]. *Hibiscus sabdariffa* could, in fact, be considered an optimal source of bioactives for the treatment of a multitude of disorders and illnesses [27, 28, 31, 32, 34–36].

Phenolics are used in preventive and therapeutic-based management of obesity. They are among the most promising polyphenols which are in general molecularly diverse and of multifactorial nature. The dietary intervention employing these phenolic compounds is promising [20, 37–43]. In experiments accomplished using cells and animal models, peroxisome proliferator-activated receptors (PPAR) and AMP-activated protein kinase (AMPK) and other compounds were demonstrated to be potentially targeted by *Hibiscus sabdariffa* polyphenols [32, 44, 45]. Consequently, it can be hypothesized that a preventive antiobesity therapy could be realized by manipulating thermogenesis [29]. *H. sabdariffa*-derived polyphenols improve the status of diverse antiobesity-related conditions based on studies accomplished on cells, animal models, and humans. *H. sabdariffa* polyphenols function as possible ligands for different digestive and metabolic enzymes [29]. They can exert an enzyme inhibitory effect regarding amylase and pancreatic lipase and that could be associated with weight loss [46, 47].

Polyphenols were also suggested to be antioxidant, scavengers of peroxyl radicals, and superoxides.

They are generally of importance in regulation of excess in reactive oxygen species (ROS) and consequently involved in obesity-related conditions through ameliorating obesity-linked oxidative stress [23–33]. These antioxidant virtues of polyphenols have also protective functions regarding chronic inflammation [48, 49]. Polyphenols were demonstrated to be regulators of transcription factors (i.e., AMPK, PPARs, and SREBP-1c) implicated in lipid and glucose homeostatic metabolism [32, 44, 50]. The mechanisms of action of polyphenols described to follow pleiotropic functioning fashion might pass through signal transduction pathways including CCAAT/enhancer-binding protein *α*, the peroxisome proliferator-activated receptor *γ*, adenosine monophosphate-activated protein kinase, peroxisome proliferator activator receptor gamma activator 1-alpha, sirtuin 1, sterol regulatory element-binding protein-1c, uncoupling proteins 1 and 2, and NF-*κ*B, which are important for the regulation of adipogenesis, antioxidant, and anti-inflammatory responses [3].

Alkaloids represent one of the most diverse groups of herbal secondary metabolites. Alkaloids are found and produced (even though at minimal concentrations) in almost all plants in addition to various organisms like animals, fungi, and bacteria. Alkaloids possess variable pharmacological effects even though most of them are toxic to the other species (Figure 1). Structurally, they are highly diverse and cannot be unified in a clear-cut chemical group, contrary to most phytochemicals. Alkaloids influence clearly metabolism. *Cytisus scoparius*, for example, was found to increase metabolism and enhance the elimination of fluids in obese individuals. Caffeine, ephedrine, and capsaicin were shown to manifest antiobesity effects by enhancing lipolysis and thermogenesis in addition to reducing appetite. Pharmacological effects of alkaloids encompass almost every body system: caffeine exerts a stimulating role on the nervous system; ricinine possesses toxicity effects on the digestive system with severe irritation in addition to diarrhea and vomiting (typical manifestations). Alkaloids target also blood vessels. A famous example is reserpine extracted from *Rauwolfia vomitoria*; it can lower blood pressure and is consequently used as a hypertension treatment [3, 23, 24, 47, 51].

## 2. Phytochemicals Used for the Management of the Major Basic Antiobesity Mechanisms

**Figure 2:**
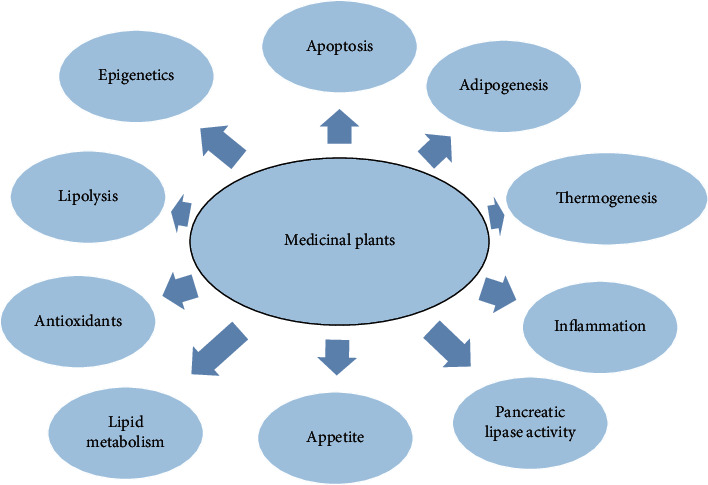
Potential action mechanisms by which diet and antioverweight and antiobesity medicinal plants exert their preventive/therapeutic action.

Medicinal plants and plant products represent the oldest and most widespread form of medicine. At least 25% of the active compounds in currently prescribed synthetic drugs were first identified in plant sources. Dissatisfaction with the high costs and potentially hazardous side effects of synthetic drugs has resulted in a widespread purchasing and exploring the utilization of herbal-based remedies than before. Several plants like poppy, foxglove, cinchona, willow, back seeds, aloe, and garlic have been verified as medicinally beneficial through repeated *in vitro* as well as clinical testing. A number of plant extracts like green tea, garlic compounds, black seed active compounds, fenugreek extracts, and conjugated linoleic acid (CLA) were shown to either exhibit antiobesity effects or have direct effects on adipose tissue (Figure 2) [50–53].

As aforementioned, among the important plant-derived active components and their weight-reducing effects are polyphenols, which represent a universal group of herbal secondary metabolites phytochemicals [51]. They are prevailing in fruits, vegetables, cereals, and legumes besides many medicinal herbs. Several *in vitro* animal and clinical studies demonstrated the antiobesity characteristics of polyphenolic compounds like grape seed proanthocyanidin extract, xanthohumol, genistein, daidzein, cyanidin, apigenin, luteolin, kaempferol, myricetin, quercetin, and epigallocatechin gallate (EGCG). Similarly, carotenoids studies aiming to investigate the effects on lipid metabolism have been accomplished using coumarin derivatives such as esculetin, fucoxanthin, and phytoalexins such as resveratrol. Other bioactive components of food possessing antiobesity properties include phytosterols, polyunsaturated fatty acids, and organosulfur compounds [3, 23, 54, 55]. The following core mechanisms are employed to obtain weight-decreasing virtues from medicinal plants and isolated phytochemicals (Table 1): (A) decreasing of appetite; (B) inhibiting pancreatic lipase activity; (C) enhancing thermogenesis and lipid metabolism; and (D) increasing of satiety and (E) epigenetic mechanisms.

### 2.1. Control of Appetite

Several plants possessing antipancreatic lipase characteristics are summarized in Table 1. Among the studied phytochemicals demonstrated to exert antiobesity effects via reducing appetite are the following with their corresponding plant source: epigallocatechin gallate, EGCG (*Camellia sinensis*), steroidal glycoside (*Hoodia gordonii* and *Hoodia Pilifera*), saponins (*Panax ginseng*), hydroxycitric acid, HCA (*Garcinia cambogia*), lectins (*Phaseolus vulgaris*), ephedrine (*Ephedra* species). This mechanism of appetite reduction is considered as the first line in the regulation of body weight and encompasses multifactorial effects of a complex of more than 40 orexigenic and anorexigenic hormones, neuropeptides, and enzymes, alongside their corresponding receptors. These mediators are synthesized in the hypothalamus beside the digestive tract, liver, and adipose tissue. In the short term, the appetite could be regulated by neural and hormonal signaling from the gastrointestinal tract, which represents the largest endocrine organ in the body and is thought to play an essential role in regulating appetite via the secretion of multiple regulatory peptide hormones. The peptide hormone ghrelin, an orexigenic hormone produced mainly in the stomach, binds to the growth hormone secretagogue receptor which is predominantly expressed in the hypothalamus; the consequent antagonisms via brain stem ghrelin might reduce the increased appetite. Hence, peptide hormones of the gastrointestinal tract are considered potential targets for the treatment of obesity. Interestingly, there is a direct relationship between the fatty acid synthesis and appetite. Fatty acid synthase is consequently directly correlated with appetite. Therefore, the inhibition of fatty acid synthase would be a potential therapeutic target to enhance weight loss through appetite suppression. This is supported by experiments on test animals, where fatty acid synthase inhibitors could reduce food intake and body weight, presumably by reducing appetite. In this respect, a growing list of medicinal plants and their extracts are demonstrated to decrease or even inhibit the activity of fatty acid synthase and hence decrease appetite [3, 23, 56, 57].

### 2.2. Pancreatic Lipase Activity

Inhibition of enzymes involved in digestion and absorption of nutrients, namely, carbohydrates, and more importantly lipids represent an efficient strategy for prevention of weight gain and also for loss of the unwanted mass. Decreasing the digestion and absorption of fats represents an efficient target in decreasing the body weight and the undesirable weight gain consequences of the high-energy food intake. An important target enzyme is the pancreatic lipase, which catalyzes the cleavage of dietary triglycerides to glycerol and fatty acids. Hydrolysis of 50–70% of total dietary fats takes place through the action of this enzyme. After cleavage, these fatty acids are integrated into bile acid-phospholipid micelles to be absorbed through the small intestines brush borders to ultimately enter the peripheral circulation as chylomicrons. The inhibition interference of pancreatic lipase could give satisfactory results through decreasing lipids hydrolysis that leads to the useful reduction in the utilization and absorption of the ingested lipids. Extensive investigations explored such inhibitory virtues (on pancreatic lipase) of many medicinal plants, herbs, their extracts, and their derived active phytochemicals. Many medicinal plants were demonstrated to possess pancreatic lipase inhibitory virtues. Several plants possessing antipancreatic lipase characteristics are summarized in Table 1. Here is a display of some examples of secondary metabolites phytochemicals with their corresponding source plant(s). These phytochemicals were demonstrated to exert their desirable effects in decreasing lipid absorption and body weight gain, namely, by inhibiting the pancreatic lipase: catechins and saponins (epigallocatechin gallate, EGCG) from green tea, polyphenols (e.g., mangiferin and catechins) and condensed tannins from *Salacia reticulate*, punicalagin, ellagic acid, and anthocyanins from pomegranate, rosmarinic acid and carnosic acid from rosemary, and proteins and isoflavones from soybean [3, 58, 59].

In this respect, animals supplemented with *H. sabdariffa* polyphenolic extracts excreted significantly large quantities of fats (essentially palmitic and oleic acids) in feces. It was concluded that such polyphenols can lead to an amelioration of obesity-associated metabolic conditions similar to that obtained using orlistat drug (currently used as digestion inhibitor). It was hypothesized that polyphenols induced inhibition of digestion enzymes like the pancreatic lipase. The underlying molecular mechanisms are still to be elucidated, even though it was plausibly forwarded that polyphenols interact directly with the enzymatic catalytic sites [26, 60, 61].

### 2.3. Thermogenesis and Lipid Metabolism

Our bodies have an autonomic, nervous, endocrine, and metabolic equilibrium that functions in coordination to maintain the body mass namely body lipids at a given level that is considered as a central nervous system given “ideal.” Unsurprisingly, after a successful weight reduction, this equilibrium yields 80% weight regain as related to the preloss body fatness. An essential component of energy expenditure is represented by brown adipose tissue which is responsible for extracting energy in the form of heat, a process known as thermogenesis. The above-mentioned equilibrium necessitates “adaptive thermogenesis” which is responsible for creating the ideal situation for weight regain and is operant in both lean and obese individuals including those attempting to sustain reduced body weight. The weight loss is therefore difficultly sustained due to an “opposition” to maintain it. This opposition is mainly attributed to the adipocyte-derived hormone “leptin.” Mitochondria function essentially in the described adaptive thermogenesis metabolic pathway that is regulated by PPARs *γ* coactivator-1alpha (PGC-1*α*). This metabolic pathway is responsible for the oxidation of lipids and dissipation of heat following the uncoupling of the mitochondrial electron transport chain (due to ATP production) under the effect exerted by uncoupling protein 1 (UCP1). Thermogenesis was reported to increase in the mitochondria-rich brown adipose tissue. It is also observed in white adipose tissue, which contains brown-like cells. This antiobesity beneficial thermogenesis process has been reported to be positively affected by secondary metabolites phytochemicals like capsaicin, caffeine, ephedrine, resveratrol, epigallocatechin gallate (EGCG), gingerol, and oleuropein. Such compounds have been, therefore, reasonably suggested as treatments for obesity and overweight.

Some antiobesity biochemical mechanisms underlying functioning of these phytochemicals were described. Caffeine, for instance, mediates thermogenic effects by inhibiting the phosphodiesterase-induced degradation of the intracellular cAMP. In addition, caffeine underlines decreasing food intake and consequently energy stockpiling in form of lipids. Capsaicin, which is an alkaloid, and epigallocatechin gallate, which is a flavonoid, were also demonstrated to augment thermogenesis in studies accomplished on humans. In a dose-dependent manner, capsaicin was found to stimulate catecholamine secretion from the adrenal medulla, which possesses a thermogenic effect. EGCG enhances thermogenesis by inhibiting catechol methyl-transferase, which is an enzyme responsible for the degradation of norepinephrine [3, 23, 26, 62–66].

### 2.4. Satiety

A clear antiobesity effect is obtained through increased satiety which is enhanced by the dietary fibers. The efficacy of dietary fibers in the prevention/treatment of obesity as well as obesity-related disorders has been demonstrated in several recent long-term studies. Higher satiety was clinically found to be achieved by fiber-rich diets compared to low-fiber nutrition. Fiber-containing diets are helpful to decrease the body mass index and helpful in weight loss and weight maintenance in obese individuals. Cellulose, hemicellulose, lignin, pectin, gum, mucilage, and soluble fibers are examples of dietary fibers found in many whole plant foods such wheat, corn, and rice bran which are known to have a high level of insoluble fibers. Increased satiety can be obtained by supplementing food with gel-forming fibers, such as guar gum. The effect of fibers can be attributed to slower gastric emptying. An important characteristic of dietary fibers is that they are generally not digestible by the human digestive system but may be fermented by microflora prevailing in the gut. The dietary fibers can be categorized into two groups: soluble or fermentable fibers and insoluble fibers, which can be, however, fermented by gut microflora. Soluble fibers are natural hydrogel-forming fibers and include pectin, gum, and mucilage. In contrast, insoluble fibers are structural fibers like lignin, cellulose, and some hemicelluloses. Insoluble fibers possess the characteristic of decreasing appetite and hence decreasing diet intake by exerting a hydrogel effect. This effect leads to slow absorption of energy-rich food. In case of pectin-containing meals, retarded gastric emptying in addition to satiety was demonstrated. The regulation of eat behavior is under the endocrine control of the digestive tract, where more than twenty hormones are involved. Of central importance also, the hypothalamus produces the orexins neuropeptides which are implicated in the control of food intake. Satiation has been recently linked to orexigenic and anorexigenic variations responsible for enhancing and inhibiting appetite, respectively. How these control elements are regulated by the food fibers needs to be further elucidated.

### 2.5. Targeting of Adipose Tissue

Adipogenesis occurs in adipose tissue and implicates the formation of new mature adipocytes starting with precursor cells (Figure 2). This ultimately leads to an augmentation of the size of adipocyte. Adipogenesis represents the adipocyte life cycle, and treatments that regulate both size and number of adipocytes can provide a better therapeutic approach for managing in a consequent perspective of treating obesity. The decrease in adipose tissue weight that occurs with body weight loss may be a consequence of mature fat cell loss through apoptosis and/or mobilization of lipids (lipolysis). Obesity development is of greater burden for middle-aged individuals. Elderly individuals are also susceptible and can have an augmentation of body fat content in addition to adipocytes accumulation in other tissues (notably muscle and bone marrow). The accumulation of marrow adipocytes is of special concern as it destabilizes the normal turnover of bone tissue with blood and consequently would decrease osteoblast proliferation. Therefore, treatments that lead to a downregulation of marrow adipogenesis and reduction of bone marrow adipocyte populations can have remarkable health benefits for bones. Importantly for the elderly, loss of weight is correlated with an accelerated loss of both muscle tissues and bones. That is why treatments that selectively decrease or eliminate adipocytes while maintaining and sparing muscle and bone tissues can be ultimately important in elderly people where the risks of osteoporosis and adiposity are high and should be prevented [3, 55, 67].

**Figure 3:**
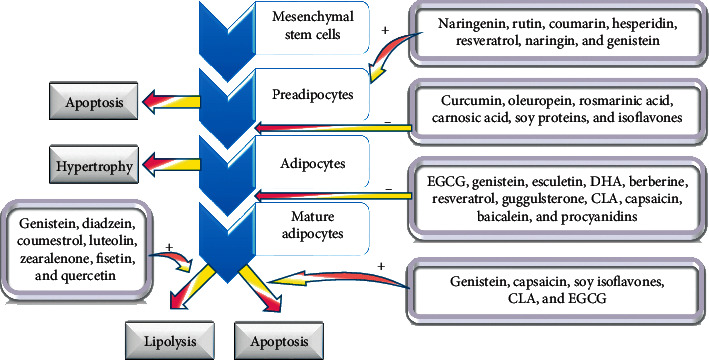
Phytochemicals their effects on adipocyte life cycle. Docosahexaenoic acid (DHA), conjugated linoleic acid (CLA), (−)−hydroxycitric acid (HCA), and (−)−epigallocatechin gallate (EGCG).

The design, development, and recommendation of antiobesity management including consumption of natural products and using drugs depend essentially on adipogenesis inhibition/apoptosis enhancement balance that targets directly the mass of the adipose connective tissue (Figure 3). Formerly, it was thought that the number of adipocytes throughout life remains constant. However, it was demonstrated recently that it is not, and the adipocyte number is subject to change under the adipogenesis/apoptosis balance. In fact, the number or size of mesenchymal stem cell-derived adipocytes or both play a continuous role in obesity throughout life.

At the cellular level, important stages of the adipocyte life cycle are of concern. This encompasses cell shape changes, arrest of growth, and clonal expansion, in addition to sophisticated sequences of alterations in gene expression leading to lipid stockpiling and ultimately to the death of cells. It has been shown that, in late stages of the differentiation of adipocytes, the mRNA levels for several enzymes are involved in triacylglycerol metabolism augment spectacularly. Examples of these key enzymes include glycerol-3-phosphate dehydrogenase, glyceraldehyde-3-phosphate dehydrogenase, and fatty acid synthase [3, 23, 68, 69].

It should be pointed out that adipose tissue is not merely metabolic but importantly represents an endocrine organ implicated in the secretion of a large number of hormones and cytokines (adipokines). Such secretions are responsible for the regulation of lipid metabolism, insulin sensitivity, glucose homeostasis, and inflammation among others. The adipocytes undergo hypertrophy in obese people which could be molecularly disturbing for the cellular functionality [70]. Consequently, there is accordingly an increase in the secretion of proinﬂammatory adipokines in the obese tissue (as IL-6, TNF-*α*, MCP-1, and vascular cell adhesion molecule-1 (VCAM-1)). This leads to a low-grade inﬂammation systemic condition including inﬁltration of macrophage in adipose tissue [71, 72]. Chronic inﬂammation and oxidative stress are interdependently associated with the obesity-associated pathogenesis [72]. Strikingly, lipid build-up in muscle, pancreas, and liver among other organs can affect IR and inﬂammation. Furthermore, liver steatosis (fats build-up), atherosclerosis, and type 2 diabetes metabolic diseases can develop [1, 73, 74].

### 2.6. Preadipocyte Apoptosis

Apoptosis is the programmed death of cells evolved initially to dispose of unwanted cells that would hinder growth and development and consequently is a natural mechanism and phenomenon essential for cellular homeostasis. Apoptosis is of special concern in the field of obesity management. Apoptosis implicates the activation of caspases through two different pathways: the death receptor pathway and the mitochondrial pathway [75]. Induction of adipocytes apoptosis employing plant-derived phytochemicals can reduce the lipids mass with an effect lasting much more than the lipolysis and lipid mobilization individually. Regulating the adipocyte life cycle efficiently can be accomplished through targeting of maturing preadipocytes by natural products (Figure 3).

Preadipocyte proliferation was found to be attenuated by a number of phytochemicals that enhance apoptosis. Apoptosis was demonstrated to be induced in preadipocytes under the cytostatic and antioxidant effect of flavonoids such as resveratrol, naringin, genistein, naringenin, rutin, hesperidin, the hot pepper-derived capsaicin, green tea polyphenol EGCG, and quercetin. This latter flavonoid is one of the most abundant fruits and vegetables' flavonoids. Apoptosis enhancement in preadipocytes takes place through pathways implicating caspase 3, Bax, and Bak activation beside downregulation of Bcl-2 and cleavage of PARP. Furthermore, preadipocyte cell cycle arrest at the G1 phase was accomplished upon treatment with phenolic acids like o-coumaric acid, m-coumaric acid, and chlorogenic acid. This effect was demonstrated to be time- and dose-dependent. Human preadipocyte apoptosis was also recently demonstrated to be promoted by CLAs and EGCG. The later treatment (with EGCG) took place in postconfluent maturing preadipocytes under insulin treatment and further investigations are needed to understand the biochemical mechanisms involved. The enhancement of apoptosis in postconfluent differentiating cells is expected to decrease ultimately the adipocytes numbers [23, 76, 77].

### 2.7. Adipocyte Apoptosis

Animal studies showed a marked reduction in body lipids mass under the effect of conjugated linoleic acid (CLA), EGCG, capsaicin, soy isoflavones, and genistein. Recent studies demonstrated that the adipocyte apoptosis under the effect of CLA could be induced by the marked increase in TNF*α* mRNA, which was clearly detected after treatment of adipocytes with uncoupling protein 2 (UCP2). The enhancement of adipocyte apoptosis by EGCG is seemingly via caspase 3, p53, protein-1, and nuclear factor kappa B (NF-*κ*B). More investigations should be conducted to elucidate the underlying mechanisms [23, 78, 79]. The apoptotic effects of EGCG, capsaicin, genistein, and ajoene are exerted via the inducing of ROS release, which enhances the activity of AMP-activated protein kinase (AMPK). The latter enzyme represents, therefore, an important target for antiobesity management and treatment. ROS were also found to be generated and implicated in the apoptotic action of ajoene in leukemic cells and similarly, ROS-mediated apoptosis could be revealed, importantly, in adipocytes [23, 80, 81].

### 2.8. Adipocytes Differentiation

A multitude of phytochemicals demonstrated interesting effects notably in attenuating adipocytes differentiation and consequently adipogenesis as well as increasing lipid mobilization (Figure 3). Capsaicin, EGCG, genistein, resveratrol, CLA, berberine, procyanidins, baicalein, esculetin, and docosahexaenoic acid (DHA) among others were shown to reduce adipocytes differentiation. Adipocytes under the effect of capsaicin, EGCG, genistein, and berberine showed a decreased expression of CCAAT/enhancer-binding protein (C/EBP*α*) and peroxisome proliferator-activated receptor (PPAR) *γ* protein transcription factors. The consequence is an attenuation effect on adipogenesis as these transcription factors are implicated in the preadipocyte growth arrest which is a prerequisite for the differentiation of adipocytes. In addition, polyunsaturated fatty acids (PUFAs) were demonstrated to decrease lipogenesis via two main mechanisms: attenuating the adipocyte differentiation late phase and decreasing the expression of the sterol regulatory element-binding proteins. Resveratrol was demonstrated to attenuate adipogenesis via increasing the expression of Sirt1 gene, which is known to enhance fat mobilization through downregulating PPAR*γ*. Finally, capsaicin, EGCG, and genistein were demonstrated to downregulate differentiation of adipocytes via an enhancement of AMP-activated protein kinase (AMPK) [23, 82–86].

### 2.9. Lipolysis

Lipolysis represents an essential process in lipid catabolic metabolism and consequently in energy homeostasis. Lipase is of core importance in lipolysis and it is activated mainly by phosphorylation. The most relevant lipolysis catalyzing enzyme is the hormone-sensitive lipase (HSL) of which activation necessitates phosphorylation. This phosphorylation is catalyzed by protein kinase A (PKA) or by G protein-coupled receptors and cyclic AMP-activated extracellular signal-regulated kinase (ERK). Many plant secondary metabolites phytochemicals were found to enhance lipolysis in adipocytes and therefore play an important role against obesity through their enhancement of phosphorylation. Coumestrol, flavonoids genistein, zearalenone, and daidzein enhance lipolysis in a dose-dependent manner in experiments conducted on rat adipocytes. Similarly, luteolin, fisetin, and quercetin stimulate lipolysis also in a dose- and time-dependent manner in rat adipocytes as well. The effect was synergistic when those phytochemicals were combined with epinephrine. Furthermore, these effective lipolytic flavonoids were also reported to be potent phosphodiesterase (PDE) inhibitors. Phosphodiesterase is an enzyme that breaks phosphodiester bonds that are found in nucleic acids as well as in cAMP which is of essential importance for lipolysis. Therefore, PDE inhibitors are expected to prolong the effects of physiological processes mediated by cAMP and hence will prolong stimulation of lipolysis. In a similar manner, long-term lipolysis in 3T3-L1 mouse adipocytes was demonstrated under the effect of increased cAMP and protein kinase A which were stimulated by grape seed-derived proanthocyanidins. Conjugated linoleic acid (CLA) was demonstrated to increase basal lipolysis in both mouse 3T3-L1 preadipocytes and human adipocytes. Docosahexaenoic acid (an omega-3 fatty acid) was revealed to enhance lipolysis when applied to mature adipocytes [23, 87–90].

## 3. Gastrointestinal Microbiota and Obesity and Phytochemicals Implications

The gastrointestinal tract is populated with microbiota in populations' ratios exhibiting a remarkable variation [91]. Interestingly, such diverse human intestinal microbiota are implicated in obesity. Alterations in the relative abundance of the human gastrointestinal lumen prominent *Bacteroidetes* and the *Firmicutes* influence obesity in animal models as well as human-based studies. Increased levels of Actinobacteria are also relevant [92, 93]. An association between the gastrointestinal canal microbiota and obesity was revealed with an impact even when transferred between experimental animals. When germ-free mice were colonized with microbiota obtained from obese animals, their total body fat significantly increased compared with colonization with microbiota from lean (nonobese) animals [94]. Dysbiosis describing alterations or imbalances of the gastrointestinal microbiota make-up and/or function was demonstrated to be implicated in the onset and/or development of obesity as well as in atherosclerosis [95] and lipid metabolism [96].

In this respect, berberine (a quaternary ammonium salt from the protoberberine group of benzylisoquinoline alkaloids) can be extracted from several medicinal plants. It is found usually in the roots, rhizomes, stems, and bark of many plants as *Mahonia aquifolium* (Oregon grape), *Berberis aristata* (tree turmeric), and *Berberis vulgaris* (barberry) among others. Berberine has recently been demonstrated to impact the intestinal microbiota composition (make-up) as well as functioning and consequently revealed antiatherosclerotic effect. A remarkable association with lipid and glucose metabolism changes as well as with anti-inflammation effect was demonstrated [97]. Food ingredients like quercetin (a bitter-flavored compound found commonly in many fruits, leaves, vegetables, grains, seeds, kale, and red onion) with their effects on the intestinal microbiota and obesity have been focused on recently. Interestingly, rats suffering from intestinal dysbiosis (due to diet high in sucrose and fats) showed a significant improvement and alleviation upon administration of quercetin [98].

Supplementing diet with quercetin could attenuate the *Firmicutes*/*Bacteroidetes* ratio and inhibit the growth of bacterial species underlying diet-enhanced obesity. Microbiota dysbiosis was reversed and obesity was improved under the supplementation with quercetin combined with resveratrol in rats having lived on high-fat diet (HFD) [99]. It has been shown that inflammation status underlines the influence of quercetin on the intestinal microflora. Mice colitis induced by *Citrobacter rodentium* bacterium is known as an animal model of inflammatory bowel disease (IBD). This colitis was shown to be alleviated in mice preadministrated with quercetin. This was attributed to the capacity of quercetin to suppress proinflammatory cytokines, for example, TNF-*α* and IL-6, besides a possible functioning of quercetin in intestinal microbiota alteration. *Bifidobacterium*, *Bacteroidetes*, and *Lactobacillus* population numbers may be enhanced in contrast with reduced *Fusobacterium* and *Enterococcus* population numbers under the influence of quercetin preadministration [100]. Quercetin-microbiota interactions could be implicated in moderating intestinal inflammation through reverting gut dysbiosis with linked enhancement of endotoxemia-mediated TLR4 pathway. Atherosclerotic lesions as well as plaques size were decreased in orally quercetin-administrated mice having lived on HFD diets. This was underlined by tuning of intestinal microbiota balance or composition [101, 102].

## 4. Epigenetics and Epigenetic Action Mechanisms

Gene expression does not depend solely on the DNA sequences but also on epigenetic appendages on the nucleotides and also on the histones. That explains “above-genetics” or the commonly and correctly coined epigenetics. These appendages are influenced by variable environmental factors including nutrition. Epigenetic mechanisms could have evolved initially to silence retroviral invasion of the genome and subsequently the epigenetic processes were coopted to control and regulate tissue differentiation [103]. These epigenetic modifications are mostly “wiped clean” and reprogrammed in mammalian early gametogenesis and early embryogenesis and later on progressively reinitiated in the function of the new environmental circumstances throughout development [104]. After fertilization in mammals, rapid demethylation of the entire paternal genome takes place, with the exclusion of heterochromatin around centromeres and some repetitive elements as well as paternally imprinted genes [105]. Contrastingly, the maternal genome is relatively slowly demethylated [106].

Among the mechanisms of epigenetics are covalent modifications of amino terminus of histone tails (e.g., methylation, acetylation, phosphorylation, and ubiquitylation) [107, 108], DNA modifications include methylation, and packaging of DNA around nucleosomes, besides chromatin folding and attachment to the nuclear matrix. The small interfering RNAs and microRNAs (si and mi RNA) are also of importance [109, 110]. The biology of adipose tissue as well as energy balance regulation has been suggested to be particularly undertaken by modifying certain miRNAs [111].

### 4.1. Inheritance of Epigenetic Modifications

Epigenetic modifications are environmentally interactive and consequently considered as plastically dynamic genomic processes that are potentially transmissible (heritable) through generations [112]. In fact, part of the epigenetic modifications can “bypass” the early developmental “wiping” of epigenetic features and can be transmitted to the next generation along with its phenotypic consequences. This can lead to an epigenetic transmission through generations in addition to the “transmission and replication” of environmental conditions and lifestyle habits including nutritive ones that will continue to produce similar if not identical epigenetic modifications transgenerationally. Consequently, the environmental-developmental register and history marks are carried in the cellular epigenome. The environmental impact on genes, genetics, epigenetics, and epigenomics is a wide field of active research and it is engendering new horizons of applied research notably in the field of nutrigenomics. The idea of epigenetics contrasts with the seemingly highly simplistic traditional principle of invalidity of acquired traits inheritance, which is prevailing in most biological classical literature. It recalls the principal idea of nature-nurture components of our genotype, epigenotype, and phenotype. Our health and diseases are, therefore, shaped not only by genes but also by genes-environment interactions and action of environment on our transmissible or heritable genes [113–116]. Changes in the epigenome during development could, therefore, be passed on to subsequent generations. Evidence obtained from rodent experiments shows that nutritional and endocrinological interventions during gestation result in phenotypic and/or epigenetic changes that persist for at least the first and the second filial generations (F1 and F2) [117–120].

Inheritance or transgenerational studies show different degrees of constancy. An experiment conducted on pregnant women whose diet was supplemented with docosahexaenoic acid (DHA) which is n-3 omega-3 fatty acid leads to increasing methylation of IGF2/H19 gene in the offspring (a gene involved in the infant metabolism, besides growth, and development, etc.). In addition, IGF2/H19 methylation modifications were linked to paternal obesity and also to the risk of diabetes, overweight, and cancer in children [121]. Contrastingly, another experiment revealed that no effect on the total methylation profile in offspring was obtained upon supplementing pregnant women's diet with DHA even though 21 sex-dependent differentially methylated DNA regions and genes were identified at birth. Among these, CCK is involved in appetite regulation, and ESYT3 is involved in lipid exchange between membranes [122]. The unbalanced maternal diet was shown to be involved in the development of insulin resistance (IR) and obesity in the offspring. Furthermore, rat experiments demonstrated that maternal butyrate supplementation (NaB) leads to offspring with impaired glucose tolerance and higher insulin resistance attributed to lipogenic genes overexpression. This was associated with an increase in histone H3 (Lys9) and H3 (Lys27) acetylation in the skeletal muscle of the adult offspring. Butyrate seems to have decreased lipid metabolism and impaired insulin sensitivity in the offspring [123, 124]. In this respect, it was shown that undernutrition during development leads to subsequent metabolic disease. Human fetuses suffering from *in utero* hyperglycemia condition, whose mothers are obese, are more susceptible to translate these environmental conditions into metabolic disorders such as type 2 diabetes mellitus later on in their lives. It is important to point out that the mammalian epigenome is mostly susceptible to nutritional cues during the entire early development stages, namely, starting from even conception and extending through weaning at least [104, 125].

### 4.2. Herbal and Diet-Derive Compounds and Epigenetics

*Hibiscus sabdariffa*-derived polyphenols were demonstrated to regulate miRNA expression in hyperlipidemic mice with LDL receptor deﬁciency [32]. These epigenetic modifications are being increasingly recognized as key mechanisms of epigenetic gene regulation. Therefore, even in genetically identical individuals, individual phenotypic variability is observed alongside cellular epigenetic mosaicism. The nutritional and environmental factors have the potential to influence organisms from their fetal to adult as well as transgenerationally through epigenetic effects underlying gene regulation. Organisms exhibit tissue-specific patterns of both DNA methylation and histone modification [104, 126–129]. Curcumin and catechins among others are examples of plant-derived polyphenols capable of interacting with enzymes as well as with epigenetics modulators including DNA methyltransferases, histones acetyltransferases, deacetylases, and kinases as well as miRNA [130, 131].

**Figure 4:**
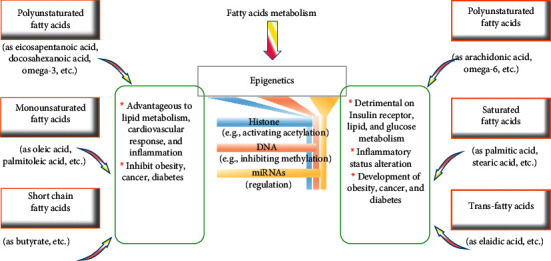
Summary of the main metabolic effects of fatty acids that can be mediated by epigenetic mechanisms. PUFA: polyunsaturated fatty acids, MUFA: monounsaturated fatty acids, SFA: saturated fatty acids, SCFA: short-chain fatty acids, EPA: eicosapentaenoic acid, DHA: docosahexanoic acid, AA: arachidonic acid, NCCD: noncommunicable chronic disease, miRNAs: noncoding microRNAs, and IR: insulin resistance [63].

Fatty acids are reported to play an important role in the regulation of gene expression through epigenetic mechanisms (Figure 3). The results on metabolism including obesity can be either positive or negative [124]. Fatty acids are capable of altering the epigenome and affecting genes implicated in decreasing insulin resistance and diabetes as well as ameliorating the metabolism of lipid and glucose [131]. The possibility of reprogramming the epigenetic parameters is promising to manage chronic disorders (like obesity). This is doable through altering lifestyle and consumption of nutrients implicated in epigenetic alterations. Among the nutrients reported to be of concern are amino acids, vitamins, minerals, methyl donors, polyphenols, fatty acids, and other phytochemicals (Figure 4) [132].

Polyunsaturated acids (PUFA) were shown to exert specific effects on DNA methylation. Omega-3 is characterized by the presence of double bond, three atoms away from the terminal methyl group, available in both animal and plant foods. Omega-6 is characterized by the presence of double bond, six atoms away from the terminal methyl group, also available also in both animal and plant foods [133]. Examples of such PUFAs encompass also arachidonic acid (AA) as well as eicosapentaenoic acid (EPA) and docosahexaenoic acid (DHA) [134]. One of the best explored fatty acids is the short-chained butyric acid which is considered a histone deacetylation inhibitor [135].

From a preventive and a treatment perspective, diet supplementation omega-3 was studied for its epigenetic antiobesity effects through a 6-month supplementation experiment on obese and overweight individuals. This investigation was culminated by demonstrating that 308 CpG sites representing 231 genes had an altered methylation profile, 286 sites among them were hypermethylated, and 22 were hypomethylated. It was demonstrated that these epigenetic alterations were relevant for pathways associated with lipid metabolism among several others (inflammation, cardiovascular signaling, type 2 diabetes, etc.) [135]. In a similar study accomplished also on obese individuals, energy-restricted diet was supplemented with omega-3-rich fish oil, leading to an increase in the methylation levels of PDK4 (Pyruvate Dehydrogenase Kinase 4) and FADS1 at many CpG sites alongside amelioration in loss of weight. This latter advantageous effect was associated also with an alteration in the methylation profile of CD36 gene (precisely in a specific CpG site). This is the gene of a membrane glycoprotein essential in lipid metabolism. Consequently, it could be involved in obesity-linked complications, for example, type 2 diabetes and glucose intolerance [134].

A study accomplished the effect exerted by omega-3 on Yupik population natives who are known to consume high levels of this fish-derived fatty acid. Twenty-seven differentially methylated CpG sites were hypothesized to decrease the expression of FAS (apoptosis antigen 1). This is expected to control and regulate the metabolism of lipid via an apoptotic pathway. Furthermore, omega-3 consumption affected the methylation profile of AHRR gene (Aryl-Hydrocarbon Receptor Repressor implicated in oxidative stress). This effect accompanied other positive effects, namely, insulin sensitivity and glucose tolerance [136]. A study conducted on Mediterranean food complemented with either nuts or extra virgin olive oil leads to hypomethylation results alongside ameliorating the expression of genes involved in the pathways of diabetes and inflammation [137]. An investigation of the role of omega-6 intake revealed a positive correlation association with body mass index (BMI), truncal fat, and waist circumference in women. TNF-*α* promoter was hypermethylated under the effect of omega-6 intake [138].

Oleic acid (OA), a monounsaturated fatty acid (MUFA), induced a remarkable hypomethylation and consequently an enhanced expression pattern in cultured human THP-1 monocytes compared with AA which is PUFA. Furthermore, that was associated with amelioration in the inflammatory profile. Subtype and dose influence drastically influence the epigenetic effects of both MUFA and PUFA. Oleic acid, originating principally from vegetables and olive oil among other oils, is highly advantageous epigenetically in processes related to obesity, type 2 diabetes, and atherosclerosis [139].

Insulin resistance cellular model (human-urine derived podocyte-like epithelial cells, HUPECs) under high doses of palmitate (SFA) and Sprague-Dawley rat males under high-fat diet, displayed a clear fatty acid-induced memory. That could be attributed to an altering of the levels of histone methylation (H3K36me2 and H3K27me3) with an activity stimulating effect, namely, on the FOXO1 promoter. Palmitate favored insulin resistance induced gluconeogenesis and hyperglycemia persistently (postlipid normalization lasting effect), which represents a kind of cellular metabolic memory [140].

Genome-wide speaking, the effects of palmitate on DNA methylation and mRNA expression in human pancreatic islets were studied. The results were displayed as DNA methylation modifications (either increase or decrease) in different regions (intergenic sequences, core gene, 3'UTR, 5'UTR, and proximal as well as distal transcription start sites TSS sequences). DNA methylation was modified in 290 genes; 73 among them were associated with the body mass index (BMI). Palmitate also affected the expression of 1860 genes encompassing genes involved in type 2 diabetes (GLIS3, HNF1B, TCF7L2, and SLC30A8) and genes implicated in fatty acids metabolism, glycolysis, and gluconeogenesis [124, 141, 142].

In studies accomplished on women using one-to-one oleate (MUFA)-palmitate (SFA) combinations, there was an increase in DNA methylation regarding PPAR *δ* expression in human skeletal muscle cells (HSkMc) in both lean and severely obese women. The mentioned increase in methylation of the same gene was to a less extent in obese women, suggesting that the methylation epigenetic alterations are affected by the degree of obesity in an environmental-specific manner [143]. Stearate and palmitate increased the methylation of peroxisome proliferator-activated receptor gamma (PPARG) as well as IL-4 levels in murine macrophages. The proinflammatory effects of these saturated fatty acids are hypothesized to be influenced by that hypermethylation, and this was found to contribute to insulin resistance in obesity [144]. The effects of proinflammatory and metabolic aberrations harmful effects of some saturated fatty acids (e.g., stearic and palmitic) were further investigated. Their influences on insulin resistance, type 2 diabetes, hyperglycemia, lipotoxicity, dysregulation of lipid metabolism leading to lipid build-up, and obesity seem to be associated with modifications in histone acetylation and DNA methylation epigenetic mechanisms [124, 141–144].

Short-chain fatty acids are fatty acids with fewer than six carbon atoms (SFA) and are produced by microbial fermentation and absorbable in the large intestine [145]. They can be responsible for epigenetic profile modifications and consequently alteration of expression of genes relevant to cancer, glucose homeostasis, insulin sensitivity, and lipid metabolism. An example of these short-chain fatty acids is sodium butyrate (NaB) which is reported to inhibit histone deacetylases (HDAC) activity [124, 146–148]. The just above-mentioned HDAC inhibition was validated upon supplementation of juvenile diabetic rats with NaB. This inhibition is correlated with a reduction in glucose and Hba1c leading to insulin sensitivity and decreased risk of developing diabetes [149].

Using C57BL/6J mice model under a high-fat diet, the advantageous NaB antidiabetes effect was also validated in parallel to augmenting type-1 fiber ratio, ameliorating muscular acylcarnitine profile, and improving insulin sensitivity in addition to protective antiobesity and sustained adiposity and body mass (weight gain was inhibited) [150]. Contrastingly, chicken body weight responded positively to NaB under mediation of epigenetic alterations including histone hyperacetylation [151]. The effect of NaB in Chinese hamster ovary (CHO) cells was either hypomethylation of genes implicated in cell cycle, signaling and apoptosis pathways, or hypermethylation in genes involved in RNA processing and protein transport. Regarding the genes involved in the differentiation, RNA metabolism, and protein biosynthesis, both hypo- and hypermethylation effects were displayed. The affected gene regions are hypothesized to be regulatory sequences intimately associated with the mentioned cellular responses to butyrate [152]. NaB supplementation enhanced hyperacetylation of histones in bovine cells and that was underlined, presumably, by the inhibition of histone deacetylases (HDAC) among several other modifications of genes implicated in cell growth and cycle, differentiation, apoptosis, and energy metabolism [153].

Several other studies demonstrated and confirmed the role of butyrate in increasing histone acetylation in enhancing NF-*κ*B, proinflammatory response, cell proliferation, and differentiation in addition to cytokine/chemokine expression [154]. NaB is also capable of altering the expression of androgen receptors in prostate cancer cells via an enhanced H4 (Lys8) and H4 (Lys12) acetylation, favoring the suppression of tumor growth as a protective function of butyrate [155]. Similar protective functioning of butyrate (NaB) was also demonstrated in human gastric cancer cells, and that was underlined by inducing demethylation and histone modifications at the promoter region of SFRP1/2. It was also hypothesized that, by employing such mechanisms, NaB could enhance caspase activation and apoptosis [156].

Butyrate functions are multiple but seem inconsistently varying: from plasma glucose reduction, improved insulin sensitivity, and glucose homeostasis, sustaining body weight and adiposity to other less advantageous effects like insulin resistance (IR) and lipid build-up. Further investigations are needed to elucidate with discrimination the effects alongside the underlying mechanisms including the epigenetic ones in a preventive and therapeutic perspective regarding chronic and metabolic disorders [124].

It has become well known that trans-fatty acids underline metabolic disorders through epigenetics, at least partially. miRNA especially those associated with HDL (HDL-carried miRNA) were altered under the effect of intake of industrial trans-fatty acids in humans. These modern lifestyle transformed fatty acids were also found to contribute to 13 HDL-carried miRNA in the plasmatic miRNA pool. Altered miRNA is implicated in lipid metabolism and extracellular matrix receptor interaction. Presumably, miRNA mediates, therefore, the regulation of the plasma lipid metabolism [157]. The epigenetic effect of the industrial transformed fatty acids was found to pass on to the next generation (transgenerational). An example is elaidic acid supplemented to either pregnant or lactating C57BL/6 mice. Both underwent induction of global methylation in the adipose tissues of 3-month aged offspring and that correlated with adipose tissue build-up and consequently gain of weight. It was shown that elaidic acid induced methylation in cultured human THP-1 monocytes in an inverse dose-effect relationship (i.e., hypermethylation under low concentrations versus hypomethylation under high concentrations of elaidic acid). Expression of genes underlying proinflammatory and adipogenic profiles was influenced in parallel to DNA methylation. This suggests that elaidic fatty acid affects gene expression potentially through epigenetic mechanisms. Elaidic acid targets potentially regulatory elements (in the gene core body or in the intergenic zones) [158–164].

## 5. Conclusions

Obesity represents an emerging global societal, medical, and psychological burden leading to many complications including morbid and even lethal consequences. Some synthetic antiobesity drugs are in use; nevertheless, the secondary metabolites phytochemicals offer a safer and potentially more efficient and natural manner to combat the global obesity epidemic. Several examples of these phytochemicals with possible functioning mechanisms are displayed in this review, which is not pretending to be an extensive catalog but an enhancer for further investigations of more potential plant species, more phytochemicals with their mode, and mechanisms of functioning as well as more regarding the effective and safe doses and usage. These phytochemicals are to be used either alone or as complements of the available drugs.

This review discussed the combat against obesity and reduction of weight using medicinal plants, which is accomplishable through control of appetite, inhibition of pancreatic lipase activity, enhancement of thermogenesis and lipid metabolism as well as increasing of satiety. In addition, important pathways for treating obesity should take the following relevant points into consideration. Adipose tissue can be reduced via inhibiting the growth of adipocytes or through apoptosis. Enhancements of apoptosis and reduction of adipogenesis at different stages of the adipocyte life cycle in addition to promotion of lipolysis are also important regarding antiobesity approaches.

Enhancement of apoptosis and lipolysis and consequently reduction in lipid build-up could be obtained under the effect of herbal-derived bioactive compounds. The underlying signaling pathways are complexly interconnected. Therefore, employment of multiple natural products rather than synthetic drugs in the management and treatment of obesity is promising. This can be achieved through additive or synergistic actions of various natural compounds functioning on adipocytes at several targets in the various life cycles and at several molecular, cellular, and metabolic levels (adipogenesis, apoptosis, lipolysis, etc.).

In experiments accomplished on humans, various experimental designs, duration, individual study subjects' variations, phytochemicals forms, and extraction procedures among others are essential reasons responsible for the inconsistent results regarding the antiobesity virtues of the phytochemicals under study. Discrepancies between preclinical results and the inconsistent and inconclusive clinical results should be ameliorated in future investigations. In this respect, randomized controlled experimental design regarding phytochemicals assays can be helpful.

Nutriepigenomics, an emerging research domain, can be insightful in understanding the detrimental impact of the modern lifestyle on many noncommunicable chronic diseases including obesity. Importantly, this new scientific domain can also provide epigenetic solutions through modification of the lifestyle including nutritive habits, namely, focusing on natural phytochemicals-based nutrition. This represents potentially an efficient novel preventive and therapeutic lifestyle characterized by its safe aspect, provided to be sure that there are no side effects of “reasonable” dietary intake. The transient, reversible, but heritable behavior of epigenetic alterations regarding obesity can provide large obesity management horizons.

Fine-tuning of the statuses of the diverse epigenetic components (including methylation, acetylation, miRNAs, phosphorylation, ubiquitylation, DNA, and chromatin packaging) by active natural compounds, namely, phytochemicals is a promising strategy for the management of noncommunicable chronic conditions like obesity. Handling of obesity necessitates reversing the alterations and aberrations in our epigenetic and epigenomic landscape which are products of the ever less natural westernized modern lifestyle and nutrition. Hopefully, that is doable using integral management including a healthy diet like the natural phytochemicals and nutrition ingredients that are started to be explored in this present review. Let your healthy diet impact not only your health but also your own epigenome and, importantly, that of your children.

## Figures and Tables

**Table 1:** Antiobesity medicinal plants and their targets.

Plant name	Part used	Pancreatic lipase	Adipocyte differentiation	Appetite	Thermogenesis	Epigenetics mechanisms
*Adonis palaestina*	Aerial parts	√	—	√	—	—
*Aframomum melegueta*	Seeds	√	—	—	—	—
*Allium sativum*	Bulbs	—	√	—	—	√
*Alpinia zerumbet*	Seeds	√	—	—	—	√
*Salvia miltiorrhiza*	Root and rhizome	√	—	√	—	√
*Anchusa azurea*	Flowers	√	—	—	—	√
*Arachis hypogaea*	Fruits	—	—	—	√	√
*Asparagus acutifolius*	Stems	√	—	—	—	—
*Bergenia crassifolia*	Rhizomes	√	—	—	—	—
*Camellia sinensis*	Leaves	√	√	√	√	√
*Capsicum annuum*	Fruits	—	√	—	√	—
*Carthamus oxyacantha*	Aerial parts	√	—	—	—	—
*Cassia angustifolia*	Leaves	√	—	—	—	√
*Castanea crenata*	Staminate flower	√	—	—	—	—
*Cichorium intybus*	Leaves	√	—	—	—	√
*Cinnamomum zeylanicum*	Derms	√	—	—	—	√
*Citrus aurantium*	Fruits	—	—	√	√	√
*Coffea arabica*	Bean	—	—	—	√	√
*Coleus forskohlii*	Roots	—	—	—	—	√
*Cornus officinalis*	Fruits	√	—	—	—	—
*Curcuma longa*	Rhizomes	—	√	—	—	√
*Cynometra cauliflora*	Leaves	√	—	—	—	—
*Dioscorea nipponica*	Roots	√	—	—	—	—
*Diplotaxis tenuifolia*	Leaves	√	—	—	—	√
*Eleusine indica*	Aerial part	√	—	√	—	—
*Ephedra species*	Branches	—	—	—	—	—
*Eucalyptus galbie*	Leaves	√	—	—	—	—
*Euonymus alatus*	Roots	√	—	—	—	—
*Fagonia arabica*	Aerial parts	√	—	—	—	—
*Ferula asafoetida*	Resin	√	—	—	—	—
*Ficus carica*	Leaves	√	—	—	—	—
*Foeniculum vulgare*	Leaves and seeds	√	—	√	—	√
*Garcinia cambogia*	Fruits	—	√	√	—	√
*Geranium nepalense*	Whole grass	√	—	—	—	√
*Ginkgo biloba*	Leaves	√	—	—	—	—
*Glycine max*	Beans	—	—	√	—	√
*Hoodia gordonii*	Stems	—	—	√	—	—
*Hoodia pilifera*	Stems	—	—	√	—	—
*Humulus lupulus*	Hops	—	√	—	—	√
*Hypericum perforatum*	Aerial parts	√	—	√	—	—
*Juglans mandshurica*	Fruits	√	—	—	—	√
*Malva nicaeensis*	Aerial parts	√	—	—	—	—
*Mangifera indica*	Leaves and stem	√	—	—	—	—
*Melastoma candidum*	Aerial part	√	—	—	—	—
*Mentha spicata*	Leaves	√	—	—	—	—
*Millettia reticulata*	Rattan cane	√	—	—	—	
*Moringa stenopetala*	Leaves	√	—	—	—	√
*Morus alba*	Leaves	√	—	—	—	—
*Myristica fragrans*	Mace	√	—	—	—	—
*Myrtus communis*	Leaves	√	—	—	—	√
*Nigella sativa*	Seeds	√	—	—	—	√
*Olea europaea*	Leaves	—	—	—	√	—
*Ononis natrix*	Aerial parts	√	—	—	—	—
*Origanum syriaca*	Aerial parts	√	—	—	—	—
*Origanum vulgare*	Stem and leaves	√	—	—	—	√
*Orixa japonica*	Whole plants	√	—	—	—	—
*Palm oil*	Fruits	—	√	—	—	√
*Panax ginseng*	Roots	—	√	√	—	—
*Papaver rhoeas*	Leaves	√	—	—	—	—
*Paronychia argentea*	Aerial parts	√	—	—	—	—
*Passiflora nitida*	Leaves	√	—	—	—	—
*Phaseolus vulgaris*	Beans	—	—	√	—	√
*Phyla nodiflora*	Whole plant	√	—	—	—	—
*Pimpinella anisum*	Seeds	√	—	—	—	—
*Pinus koraiensis*	Pine nut		—	√	—	—
*Pistacia vera*	Fruits hall	√	—	—	—	—
*Portulaca oleracea*	Leaves	√	—	—	—	—
*Pyrus pyrifolia*	Bark and leaf	√	—	—	—	—
*Raphanus raphanistrum*	Leaves	√	—	—	—	—
*Reseda alba*	Aerial parts	√	—	—	—	—
*Robinia pseudoacacia*	Beans	—	—	√	—	—
*Rosa damascena*	Flowers	√	—	—	—	—
*Rosmarinus officinalis*	Leaves	√	√	—	—	—
*Rubi fructus*	Fruits	√	—	—	—	—
*Salicis radicis Cortex*	Bark	√	—	—	—	—
*Salvia spinosa*	Aerial parts	√	—	—	—	—
*Shorea roxburghii*	Bark	√	—	—	—	—
*Silybum marianum*	Seeds	—	√	—	—	√
*Smyrnium olusatrum*	Leaves	√	—	—	—	—
*Sonchus asper*	Leaves	√	—	—	—	—
*Sonchus oleraceus*	Leaves	√	—	—	—	—
*Spilanthes acmella*	Flower buds	√	—	—	—	—
*Trigonella foenum-graecum*	Seeds	√	—	—	—	—
*Urtica urens*	Aerial parts	√	—	—	—	—
*Vitis vinifera*	Fruits/red wine	—	—	—	√	√

## Data Availability

The data used to support the findings of this study are available from the corresponding author upon request.

## References

[1] Luna-Luna M., Medina-Urrutia A., Vargas-Alarcon G., Coss-Rovirosa F., Vargas-Barron J., Perez-Mendez O. (2015). Adipose tissue in metabolic syndrome: onset and progression of atherosclerosis. *Archives of Medical Research*.

[2] Saad B., Murad F., Rahman A.-U., Bian K., Bian (2019). Prevention and treatment of obesity-related cardiovascular diseases by diet and medicinal plants. *Herbal Medicine: Back to the Future, Volume 2: Vascular Health*.

[3] Saad B., Zaid H., Shanak S., Kadan S. (2017). Anti-diabetes and antiobesity medicinal plants and phytochemicals safety. *Efficacy, and Action Mechanisms*.

[4] Saad B. (2005). Greco-Arab and Islamic diet therapy: tradition, research and practice. *Arabian Journal of Medicinal and Aromatic Plants*.

[5] Kumar P., Bhandari U. (2015). Common medicinal plants with antiobesity potential: a special emphasis on fenugreek. *Ancient Science of Life*.

[6] Lumeng C. N., DelProposto J. B., Westcott D. J., Saltiel A. R. (2008). Phenotypic switching of adipose tissue macrophages with obesity is generated by spatiotemporal differences in macrophage subtypes. *Diabetes*.

[7] Osborn O., Olefsky J. M. (2012). The cellular and signaling networks linking the immune system and metabolism in disease. *Nature Medicine*.

[8] Ross R. (1999). Atherosclerosis - an inflammatory disease. *New England Journal of Medicine*.

[9] Okada-Iwabu M., Yamauchi T., Iwabu M. (2013). A small-molecule adipor agonist for type 2 diabetes and short life in obesity. *Nature*.

[10] Rise M. B., Pellerud A., Rygg L. Ø., Steinsbekk A. (2013). Making and maintaining lifestyle changes after participating in group based type 2 diabetes self-management educations: a qualitative study. *PLoS One*.

[11] Eckel R. H., Grundy S. M., Zimmet P. Z., Zimmet P. Z. (2005). The metabolic syndrome. *The Lancet*.

[12] Ahmadian M., Suh J. M., Hah N. (2013). PPAR*γ* signaling and metabolism: the good, the bad and the future. *Nature Medicine*.

[13] Leite C. E., Mocelin C. A., Petersen G. O., Leal M. B., Thiesen F. V. (2009). Rimonabant: an antagonist drug of the endocannabinoid system for the treatment of obesity. *Pharmacological Reports*.

[14] Heck A. M., Yanovski J. A., Calis K. A. (2000). Orlistat, a new lipase inhibitor for the management of obesity. *Pharmacotherapy*.

[15] Cangiano T., Dellagreca M., Fiorentino A., Isidori M., Monaco P., Zarrelli A. (2002). Effect of ent-labdane diterpenes from Potamogetonaceae on Selenastrum capricornutum and other aquatic organisms. *Journal of Chemical Ecology*.

[16] DellaGreca M., Fiorentino A., Monaco P., Previtera L., Zarrelli A. (2002). A new dimeric 9,10-dihydrophenanthrenoid from the rhizome of Juncus acutus. *Tetrahedron Letters*.

[17] Cangiano T., DellaGreca M., Fiorentino A., Isidori M., Monaco P., Zarrelli A. (2001). Lactone diterpenes from the aquatic plant Potamogeton natans. *Phytochemistry*.

[18] Falcone Ferreyra M. L., Rius S. P., Casati P. (2012). Flavonoids: biosynthesis, biological functions, and biotechnological applications. *Frontiers in Plant Science*.

[19] Cramer G. R., Urano K., Delrot S., Pezzotti M., Shinozaki K. (2011). Effects of abiotic stress on plants: a systems biology perspective. *BMC Plant Biology*.

[20] Menendez J. A., Joven J., Aragonès G. (2013). Xenohormetic and anti-aging activity of secoiridoid polyphenols present in extra virgin olive oil. *Cell Cycle*.

[21] Hooper P. L., Hooper P. L., Tytell M., Vígh L. (2010). Xenohormesis: health benefits from an eon of plant stress response evolution. *Cell Stress and Chaperones*.

[22] Kabera J. N., Semana E., Mussa A. R., Xin H. (2014). Plant secondary metabolites: biosynthesis, classification, function and pharmacological properties. *Journal of Pharmacy and Pharmacology*.

[23] Riaz M., Zia Ul Haq M., Saad B. (2016). *Anthocyanins and Human Health: Biomolecular and Therapeutic Aspect*.

[24] Verpoorte R. (1998). Exploration of nature’s chemodiversity: the role of secondary metabolites as leads in drug development. *Drug Discovery Today*.

[25] Goto T., Takahashi N., Hirai S., Kawada T. (2010). Various terpenoids derived from herbal and dietary plants function as PPAR modulators and regulate carbohydrate and lipid metabolism. *PPAR Research*.

[26] Racotta I. S., Leblanc J., Richard D. (1994). The effect of caffeine on food intake in rats: involvement of corticotropin-releasing factor and the sympatho-adrenal system. *Pharmacology Biochemistry and Behavior*.

[27] Park E.-S., Moon W.-S., Song M.-J., Kim M.-N., Chung K.-H., Yoon J.-S. (2001). Antimicrobial activity of phenol and benzoic acid derivatives. *International Biodeterioration & Biodegradation*.

[28] Pengelly A. (2004). *The Constituents of Medicinal Plants: An Introduction to the Chemistry and Therapeutics of Herbal Medicine*.

[29] Herranz-López M., Olivares-Vicente M., Encinar J. (2017). Multi-targeted molecular effects of Hibiscus sabdariffa polyphenols: an opportunity for a global approach to obesity. *Nutrients*.

[30] Beltrán-Debón R., Alonso-Villaverde C., Aragonès G. (2010). The aqueous extract of hibiscus sabdariffa calices modulates the production of monocyte chemoattractant protein-1 in humans. *Phytomedicine*.

[31] Fernández-Arroyo S., Rodríguez-Medina I. C., Beltrán-Debón R. (2011). Quantification of the polyphenolic fraction and *in vitro* antioxidant and *in vivo* anti-hyperlipemic activities of *Hibiscus sabdariffa* aqueous extract. *Food Research International*.

[32] Fernández-Arroyo S., Herranz-López M., Beltrán-Debón R. (2012). Bioavailability study of a polyphenol-enriched extract from hibiscus sabdariffa in rats and associated antioxidant status. *Molecular Nutrition & Food Research*.

[33] Herranz-López M., Fernández-Arroyo S., Pérez-Sanchez A. (2012). Synergism of plant-derived polyphenols in adipogenesis: perspectives and implications. *Phytomedicine*.

[34] Joven J., Espinel E., Rull A. (2012). Plant-derived polyphenols regulate expression of mirna paralogs mir-103/107 and mir-122 and prevent diet-induced fatty liver disease in hyperlipidemic mice. *Biochimica et Biophysica Acta (BBA) - General Subjects*.

[35] Joven J., March I., Espinel E. (2014). Hibiscus sabdariffa extract lowers blood pressure and improves endothelial function. *Molecular Nutrition & Food Research*.

[36] Rodríguez-Medina I. C., Beltrán-Debón R., Molina V. M. (2009). Direct characterization of aqueous extract ofHibiscus sabdariffausing HPLC with diode array detection coupled to ESI and ion trap MS. *Journal of Separation Science*.

[37] Serban C., Sahebkar A., Ursoniu S., Andrica F., Banach M. (2015). Effect of sour tea (*Hibiscus sabdariffa* L.) on arterial hypertension. *Journal of Hypertension*.

[38] Chin K. L., Zhen J., Qi Y. (2016). A comparative evaluation: phytochemical composition and antioxidant capacity of three roselle (*Hibiscus sabdariffa* L.) accessions. *Acta Horticulturae*.

[39] Barrajón-Catalán E., Herranz-López M., Joven J. (2014). Molecular promiscuity of plant polyphenols in the management of age-related diseases: far beyond their antioxidant properties. *Advances in Experimental Medicine and Biology*.

[40] Beltrán-Debón R., Rull A., Rodríguez-Sanabria F. (2011). Continuous administration of polyphenols from aqueous rooibos (*Aspalathus linearis*) extract ameliorates dietary-induced metabolic disturbances in hyperlipidemic mice. *Phytomedicine*.

[41] Herranz-López M., Barrajón-Catalán E., Segura-Carretero A., Menéndez J. A., Joven J., Micol V. (2015). *Lemon verbena* (*Lippia citriodora*) polyphenols alleviate obesity-related disturbances in hypertrophic adipocytes through ampk-dependent mechanisms. *Phytomedicine*.

[42] Ali F., Ismail A., Kersten S. (2014). Molecular mechanisms underlying the potential antiobesity-related diseases effect of cocoa polyphenols. *Molecular Nutrition & Food Research*.

[43] Amiot M. J., Riva C., Vinet A. (2016). Effects of dietary polyphenols on metabolic syndrome features in humans: a systematic review. *Obesity Reviews*.

[44] Wang S., Moustaid-Moussa N., Chen L. (2014). Novel insights of dietary polyphenols and obesity. *The Journal of Nutritional Biochemistry*.

[45] Halliwell B., Rafter J., Jenner A. (2005). Health promotion by flavonoids, tocopherols, tocotrienols, and other phenols: direct or indirect effects? antioxidant or not?. *The American Journal of Clinical Nutrition*.

[46] Villalpando-Arteaga E. V., Mendieta-Condado E., Esquivel-Solís H. (2013). *Hibiscus sabdariffa* L. aqueous extract attenuates hepatic steatosis through down-regulation of PPAR-*γ* and SREBP-1c in diet-induced obese mice. *Food & Function*.

[47] Yang M.-Y., Peng C.-H., Chan K.-C., Yang Y.-S., Huang C.-N., Wang C.-J. (2010). The hypolipidemic effect of *Hibiscus sabdariffa* polyphenols via inhibiting lipogenesis and promoting hepatic lipid clearance. *Journal of Agricultural and Food Chemistry*.

[48] Joven J., Micol V., Segura-Carretero A. (2014). Polyphenols and the modulation of gene expression pathways: can we eat our way out of the danger of chronic disease?. *Critical Reviews in Food Science and Nutrition*.

[49] Perez-Torres I., Ruiz-Ramirez A., Banos G., El-Hafidi M. (2013). *Hibiscus sabdariffa* Linnaeus (Malvaceae), curcumin and resveratrol as alternative medicinal agents against metabolic syndrome. *Cardiovascular & Hematological Agents in Medicinal Chemistry*.

[50] Said O., Saad B., Fulder S., Amin R., Kassis E., Khalil K. (2009). Hypolipidemic activity of extracts from *Eriobotrya japonica* and *Olea europaea*, traditionally used in the Greco-Arab medicine in maintaining healthy fat levels in the blood. *The Open Complementary Medicine*.

[51] Saad B., Said O. (2011). Herbal medicine. *Greco-Arab and Islamic Herbal Medicine: Traditional System, Ethics, Safety, Efficacy and Regulatory Issues*.

[52] Shanak S., Saad B., Zaid H. (2019). Metabolic and epigenetic action mechanisms of antidiabetic medicinal plants. *Evidence-Based Complementary and Alternative Medicine*.

[53] Said O., Fulder S., Khalil K., Azaizeh H., Kassis E., Saad B. (2008). Maintaining a physiological blood glucose level with ‘glucolevel’, a combination of four anti-diabetes plants used in the traditional Arab herbal medicine. *Evidence-Based Complementary and Alternative Medicine*.

[54] Han L.-K., Sumiyoshi M., Zheng Y.-N., Okuda H., Kimura Y. (2003). Anti-obesity action of *Salix matsudana* leaves (Part 2). Isolation of anti-obesity effectors from polyphenol fractions of *Salix matsudana*. *Phytotherapy Research*.

[55] Picard F., Kurtev M., Chung N. (2004). Sirt1 promotes fat mobilization in white adipocytes by repressing PPAR-*γ*. *Nature*.

[56] Seyedan A., Alshawsh M. A., Alshagga M. A., Koosha S., Mohamed Z. (2015). Medicinal plants and their inhibitory activities against pancreatic lipase. *Evidence-Based Complementary and Alternative Medicine*.

[57] Van Heerden F. R. (2008). *Hoodia gordonii*: a natural appetite suppressant. *Journal of Ethnopharmacology*.

[58] Birari R. B., Bhutani K. K. (2007). Pancreatic lipase inhibitors from natural sources: unexplored potential. *Drug Discovery Today*.

[59] Marrelli M., Loizzo M. R., Nicoletti M., Menichini F., Conforti F. (2013). Inhibition of key enzymes linked to obesity by preparations from mediterranean dietary plants: effects on *α*-amylase and pancreatic lipase activities. *Plant Foods for Human Nutrition*.

[60] Carvajal-Zarrabal O., Hayward-Jones P. M., Orta-Flores Z. (2009). Effect of *Hibiscus sabdariffa* L. dried calyx ethanol extract on fat absorption-excretion, and body weight implication in rats. *BioMed Research International*.

[61] Rodina A. V., Severin S. E. (2013). The role of adiponectin in the pathogenesis of the metabolic syndrome and approach to therapy. *Patologiceskaja Fiziologija i Eksperimental’naja Terapija*.

[62] Dulloo A. G. (1993). Ephedrine, xanthines and prostaglandin-inhibitors: actions and interactions in the stimulation of thermogenesis. *International journal of obesity and related metabolic disorders*.

[63] Astrup A., Breum L., Toubro S., Hein P., Quaade F. (1992). The effect and safety of an ephedrine caffeine compound compared to ephedrine, caffeine and placebo in obese subjects on an energy restricted diet: a doubleblind trial. *International Journal of Obesity*.

[64] Diepvens K., Westerterp K. R., Westerterp-Plantenga M. S. (2007). Obesity and thermogenesis related to the consumption of caffeine, ephedrine, capsaicin, and green tea. *American Journal of Physiology-Regulatory, Integrative and Comparative Physiology*.

[65] Dulloo A. G., Duret C., Rohrer D. (1999). Efficacy of a green tea extract rich in catechin polyphenols and caffeine in increasing 24-h energy expenditure and fat oxidation in humans. *The American Journal of Clinical Nutrition*.

[66] Dulloo A., Seydoux J., Girardier L., Chantre P., Vandermander J. (2000). Green tea and thermogenesis: interactions between catechin-polyphenols, caffeine and sympathetic activity. *International Journal of Obesity*.

[67] Jilka R. L. (2002). Osteoblast progenitor fate and age-related bone loss. *Journal of Musculoskeletal & Neuronal Interactions*.

[68] Gregoire F. M. (2001). Adipocyte differentiation: from fibroblast to endocrine cell. *Experimental Biology and Medicine*.

[69] Paulauskis J. D., Sul H. S. (1998). Cloning and expression of mouse fatty acid synthase and other specific mRNAs. Developmental and hormonal regulation in 3T3-L1 cells. *Journal of Biological Chemistry*.

[70] Greenberg A. S., Obin M. S. (2006). Obesity and the role of adipose tissue in inflammation and metabolism. *The American Journal of Clinical Nutrition*.

[71] Kang Y. E., Kim J. M., Joung K. H. (2016). The roles of adipokines, proinflammatory cytokines, and adipose tissue macrophages in obesity-associated insulin resistance in modest obesity and early metabolic dysfunction. *PLoS One*.

[72] Marseglia L., Manti S., D’Angelo G. (2014). Oxidative stress in obesity: a critical component in human diseases. *International Journal of Molecular Sciences*.

[73] O’Neill S., O’Driscoll L. (2015). Metabolic syndrome: a closer look at the growing epidemic and its associated pathologies. *Obesity Reviews*.

[74] Cancello R., Clément K. (2006). Review article: is obesity an inflammatory illness? role of low-grade inflammation and macrophage infiltration in human white adipose tissue. *BJOG: An International Journal of Obstetrics & Gynaecology*.

[75] Gupta S. (2001). Molecular steps of death receptor and mitochondrial pathways of apoptosis. *Life Sciences*.

[76] Hsu C.-L., Yen G.-C. (2006). Induction of cell apoptosis in 3T3-L1 pre-adipocytes by flavonoids is associated with their antioxidant activity. *Molecular Nutrition & Food Research*.

[77] Chan M. M., Fong D., Soprano K. J., Holmes W. F., Heverling H. (2003). Inhibition of growth and sensitization to cisplatin-mediated killing of ovarian cancer cells by polyphenolic chemopreventive agents. *Journal of Cellular Physiology*.

[78] Lin J., Della-Fera M. A., Baile C. A. (2005). Green tea polyphenol epigallocatechin gallate inhibits adipogenesis and induces apoptosis in 3T3-L1 adipocytes. *Obesity Research*.

[79] Tsuboyama-Kasaoka N., Takahashi M., Tanemura K. (2000). Conjugated linoleic acid supplementation reduces adipose tissue by apoptosis and develops lipodystrophy in mice. *Diabetes*.

[80] Kao Y.-H., Hiipakka R. A., Liao S. (2000). Modulation of obesity by a green tea catechin. *The American Journal of Clinical Nutrition*.

[81] Dirsch V. M., Gerbes A. L., Vollmar A. M. (1998). Ajoene, a compound of garlic, induces apoptosis in human promyeloleukemic cells, accompanied by generation of reactive oxygen species and activation of nuclear factor *κ*B. *Molecular Pharmacology*.

[82] Adams J. M., Cory S. (1998). The Bcl-2 protein family: arbiters of cell survival. *Science*.

[83] Kim H.-K., Nelson-Dooley C., Della-Fera M. A. (2006). Genistein decreases food intake, body weight, and fat pad weight and causes adipose tissue apoptosis in ovariectomized female mice. *The Journal of Nutrition*.

[84] Huang C., Zhang Y., Gong Z., Sheng X., Li Z., Zhang W. (2006). Berberine inhibits 3T3-L1 adipocyte differentiation through the PPAR-gamma pathway. *Biochemical and Biophysical Research Communications*.

[85] Sisk M. B., Hausman D. B., Martin R. J., Azain M. J. (2001). Dietary conjugated linoleic acid reduces adiposity in lean but not obese Zucker rats. *Journal of Nutrition*.

[86] Cha M. H., Kim I. C., Lee B. H., Yoon Y. (2006). Baicalein inhibits adipocyte differentiation by enhancing COX-2 expression. *Journal of Medicinal Food*.

[87] Kandulska K., Nogowski L., Szkudelski T. (1999). Effect of some phytoestrogens on metabolism of rat adipocytes. *Reproduction Nutrition Development*.

[88] Szkudelska K., Szkudelski T., Nogowski L. (2002). Daidzein, coumestrol and zearalenone affect lipogenesis and lipolysis in rat adipocytes. *Phytomedicine*.

[89] Kuppusamy U. R., Das N. P. (1992). Effects of flavonoids on cyclic AMP phosphodiesterase and lipid mobilization in rat adipocytes. *Biochemical Pharmacology*.

[90] Pinent M., Blade M. C., Salvado M. J., Arola L., Ardevol A. (2005). Intracellular mediators of procyanidin-induced lipolysis in 3T3-L1 adipocytes. *Journal of Agricultural and Food Chemistry*.

[91] Moore D. D., Kato S., Xie W. (2006). International Union of Pharmacology. LXII. The NR1H and NR1I receptors: constitutive androstane receptor, pregnene X receptor, farnesoid X receptor alpha, farnesoid X receptor beta, liver X receptor alpha, liver X receptor beta, and vitamin D receptor. *Pharmacological Reviews*.

[92] Ley R. E., Turnbaugh P. J., Klein S., Gordon J. I. (2006). Microbial ecology: human gut microbes associated with obesity. *Nature*.

[93] Turnbaugh P. J., Hamady M., Yatsunenko T. (2009). A core gut microbiome in obese and lean twins. *Nature*.

[94] Turnbaugh P. J., Ley R. E., Mahowald M. A. (2006). An obesity-associated gut microbiome with increased capacity for energy harvest. *Nature*.

[95] Arumugam M., Raes J., Pelletier E. (2011). Enterotypes of the human gut microbiome. *Nature*.

[96] Jonsson A. L., Bäckhed F. (2017). Role of gut microbiota in atherosclerosis. *Nature Reviews Cardiology*.

[97] Wu M., Yang S., Wang S. (2020). Effect of berberine on atherosclerosis and gut microbiota modulation and their correlation in high-fat diet-fed ApoE-/- mice. *Frontiers in Pharmacology*.

[98] Etxeberria U., Arias N., Boque N. (2015). Reshaping faecal gut microbiota composition by the intake of trans-resveratrol and quercetin in high-fat sucrose diet-fed rats. *Journal of Nutritional Biochemistry*.

[99] Zhao L., Zhang Q., Ma W. (2017). A combination of quercetin and resveratrol reduces obesity in high-fat diet-fed rats by modulation of gut microbiota. *Food & Function*.

[100] Lin R., Piao M., Song Y. (2019). Dietary quercetin increases colonic microbial diversity and attenuates colitis severity in *Citrobacter rodentium*-infected mice. *Frontiers in Microbiology*.

[101] Nie J., Zhang L., Zhao G., Du X. (2019). Quercetin reduces atherosclerotic lesions by altering the gut microbiota and reducing atherogenic lipid metabolites. *Journal of Applied Microbiology*.

[102] Shin S., Yuuka M. (2020). Modulation of chronic inflammation by quercetin: the beneficial effects on obesity. *Journal of Inflammation Research*.

[103] Matzke M. A., Mette M. F., Matzke A. J. (2000). Transgene silencing by the host genome defense: implications for the evolution of epigenetic control mechanisms in plants and vertebrates. *Plant Molecular Biology*.

[104] Peter D., Gluckman Mark A., Hanson Tatjana B., Low F. M., Beedle A. S. (2009). Epigenetic mechanisms that underpin metabolic and cardiovascular diseases. *Nature Reviews Endocrinology*.

[105] Reik W. (2007). Stability and flexibility of epigenetic gene regulation in mammalian development. *Nature*.

[106] Nafee T. M., Farrell W. E., Carroll W. D., Fryer A. A., Ismail K. M. (2008). Epigenetic control of fetal gene expression. *BJOG: An International Journal of Obstetrics & Gynaecology*.

[107] Branco M. R., Oda M., Reik W. (2008). Safeguarding parental identity: DNMTl maintains imprints during epigenetic reprogramming in early embryogenesis. *Genes & Development*.

[108] Keating S. T., El-Osta A. (2015). Epigenetics and metabolism. *Circulation Research*.

[109] Zhang T., Cooper S., Brockdorff N. (2015). The interplay of histone modifications- writers that read. *EMBO Reports*.

[110] Wendt A., Esguerra J. L., Eliasson L. (2018). Islet microRNAs in health and type-2 diabetes. *Current Opinion in Pharmacology*.

[111] Esguerra J. L. S., Nagao M., Ofori J. K., Wendt A., Eliasson L. (2018). MicroRNAs in islet hormone secretion. *Diabetes, Obesity and Metabolism*.

[112] Rottiers V., Naar A. M. (2012). MicroRNAs in metabolism and metabolic disorders. *Nature Reviews Molecular Cell Biology*.

[113] Bollati V., Baccarelli A. (2010). *Environmental Epigenetics. Heredity.*.

[114] Dana C. D. (2008). The agouti mouse model: an epigenetic biosensor for nutritional and environmental alterations on the fetal epigenome. *Nutrition Reviews®*.

[115] Dana C. D., Dale H., Randy L. J. (2007). Maternal nutrient supplementation counteracts bisphenol A-induced DNA hypomethylation in early development. *Proceedings of the National Academy of Sciences*.

[116] Jirtle R. L., Skinner M. K. (2007). Environmental epigenomics and disease susceptibility. *Nature Reviews Genetics*.

[117] Drake A. J., Walker B. R., Seckl J. R. (2005). Intergenerational consequences of fetal programming by in utero exposure to glucocorticoids in rats. *American Journal of Physiology - Regulatory, Integrative and Comparative Physiology*.

[118] Burdge G. C., Slater-Jefferies J., Torrens C., Phillips E. S., Hanson M. A., Lillycrop K. A. (2007). Dietary protein restriction of pregnant rats in the F0 generation induces altered methylation of hepatic gene promoters in the adult male offspring in the F1 and F2 generations. *British Journal of Nutrition*.

[119] Gluckman P. D., Hanson M. A., Beedle A. S. (2007). Non-genomic transgenerational inheritance of disease risk. *Bioessays*.

[120] Jimenez-Chillaron J. C., Isganaitis E., Charalambous M. (2009). Intergenerational transmission of glucose intolerance and obesity by in utero undernutrition in mice. *Diabetes*.

[121] Lee H.-S., Barraza-Villarreal A., Biessy C. (2014). Dietary supplementation with polyunsaturated fatty acid during pregnancy modulates DNA methylation at IGF2/H19 imprinted genes and growth of infants. *Physiological Genomics*.

[122] Van Dijk S. J., Zhou J., Peters T. J. (2016). Effect of prenatal DHA supplementation on the infant epigenome: results from a randomized controlled trial. *Clinical Epigenetics*.

[123] Huang Y., Gao S., Chen J., Albrecht E., Zhao R., Yang X. (2017). Maternal butyrate supplementation induces insulin resistance associated with enhanced intramuscular fat deposition in the offspring. *Oncotarget*.

[124] González-Becerra K., Ramos-Lopez O., Barrón-Cabrera E. (2019). Fatty acids, epigenetic mechanisms and chronic diseases: a systematic review. *Lipids in Health and Disease*.

[125] Boney C. M., Verma A., Tucker R., Vohr B. R. (2005). Metabolic syndrome in childhood: association with birth weight, maternal obesity, and gestational diabetes mellitus. *Pediatrics*.

[126] Yagi S., Hirabayashi K., Sato S. (2008). DNA methylation profile of tissue dependent and differentially methylated regions (T-DMrs) in mouse promoter regions demonstrating tissue-specific gene expression. *Genome Research*.

[127] Song F., Mahmood S., Ghosh S. (2009). Tissue specific differentially methylated regions (TDMr): changes in DNA methylation during development. *Genomics*.

[128] Shechter D., Nicklay J. J., Chitta R. K., Shabanowitz J., Hunt D. F., David Allis C. (2009). Analysis of histones in *Xenopus laevis*. i. a distinct index of enriched variants and modifications exists in each cell type and is remodeled during developmental transitions. *Journal of Biological Chemistry*.

[129] Nicklay J. J., Shechter D., Chitta R. K. (2009). Analysis of histones in *Xenopus laevis*. ii. mass spectrometry reveals an index of cell-type specific modifications on H3 and H4. *Journal of Biological Chemistry*.

[130] Li W., Guo Y., Zhang C. (2016). Dietary phytochemicals and cancer chemoprevention: a perspective on oxidative stress, inﬂammation, and epigenetics. *Chemical Research in Toxicology*.

[131] Frigolet M. E., Gutierrez-Aguilar R. (2017). The role of the novel lipokine palmitoleic acid in health and disease. *Advances in Nutrition*.

[132] Kiec-Wilk B., Sliwa A., Mikolajczyk M., Malecki M. T., Mathers J. C. (2011). The CpG island methylation regulated expression of endothelial proangiogenic genes in response to *β*-carotene and arachidonic acid. *Nutrition and Cancer*.

[133] Kulkarni A., Dangat K., Kale A., Sable P., Chavan-Gautam P., Joshi S. (2011). Effects of altered maternal folic acid, vitamin B12 and docosahexaenoic acid on placental global DNA methylation patterns in Wistar rats. *PLoS One*.

[134] Lee C., Kim B. G., Kim J. H., Chun J., Im J. P., Kim J. S. (2017). Sodium butyrate inhibits the NF-kappa B signaling pathway and histone deacetylation and attenuates experimental colitis in an IL-10 independent manner. *International Immunopharmacology*.

[135] Tremblay B. L., Guénard F., Rudkowska I., Lemieux S., Couture P., Vohl M. C. (2017). Epigenetic changes in blood leukocytes following an omega-3 fatty acid supplementation. *Clinical Epigenetics*.

[136] Aslibekyan S., Wiener H. W., Havel P. J. (2014). DNA methylation patterns are associated with n-3 fatty acid intake in Yup’Ik people. *Journal of Nutrition*.

[137] Arpón A., Milagro F. I., Razquin C. (2018). Impact of consuming extra-virgin olive oil or nuts within a Mediterranean diet on DNA methylation in peripheral white blood cells within the PREDIMED-Navarra randomized controlled trial: a role for dietary lipids. *Nutrients*.

[138] Hermsdorff H. H., Mansego M. L., Campión J., Milagro F. I., Zulet M. A., Martínez J. A. (2013). TNF-alpha promoter methylation in peripheral white blood cells: relationship with circulating TNF*α*, truncal fat and n-6 PUFA intake in young women. *Cytokine*.

[139] Silva-Martínez G. A., Rodríguez-Ríos D., Alvarado-Caudillo Y. (2016). Arachidonic and oleic acid exert distinct effects on the DNA methylome. *Epigenetics*.

[140] Kumar S., Pamulapati H., Tikoo K. (2016). Fatty acid induced metabolic memory involves alterations in renal histone H3K36me2 and H3K27me3. *Molecular and Cellular Endocrinology*.

[141] Hall E., Volkov P., Dayeh T. (2014). Effects of palmitate on genome-wide mRNA expression and DNA methylation patterns in human pancreatic islets. *BMC Medicine*.

[142] Ishikawa K., Tsunekawa S., Ikeniwa M. (2015). Longterm pancreatic beta cell exposure to high levels of glucose but not palmitate induces DNA methylation within the insulin gene promoter and represses transcriptional activity. *PLoS One*.

[143] Maples J. M., Brault J. J., Shewchuk B. M. (2015). Lipid exposure elicits differential responses in gene expression and DNA methylation in primary human skeletal muscle cells from severely obese women. *Physiological Genomics*.

[144] Wang X., Cao Q., Yu L., Shi H., Xue B., Shi H. (2016). Epigenetic regulation of macrophage polarization and inflammation by DNA methylation in obesity. *JCI Insight*.

[145] Bergman E. N. (1990). Energy contributions of volatile fatty acids from the gastrointestinal tract in various species. *Physiological Reviews*.

[146] Arents G., Burlingame R. W., Wang B. C., Love W. E., Moudrianakis E. N. (1991). The nucleosomal core histone octamer at 3.1 a resolution: a tripartite protein assembly and a left-handed superhelix. *Proceedings of the National Academy of Sciences*.

[147] Goldberg A. D., Allis C. D., Bernstein E. (2007). Epigenetics: a landscape takes shape. *Cell*.

[148] Li R. W., Li C. (2006). Butyrate induces profound changes in gene expression related to multiple signal pathways in bovine kidney epithelial cells. *BMC Genomics*.

[149] Khan S., Jena G. B. (2014). Protective role of sodium butyrate, a HDAC inhibitor on beta-cell proliferation, function and glucose homeostasis through modulation of p38/ERK MAPK and apoptotic pathways: study in juvenile diabetic rat. *Chemico-Biological Interactions*.

[150] Henagan T. M., Stefanska B., Fang Z. (2015). Sodium butyrate epigenetically modulates high-fat diet-induced skeletal muscle mitochondrial adaptation, obesity and insulin resistance through nucleosome positioning. *British Journal of Pharmacology*.

[151] Mátis G., Neogrády Z., Csikó G. (2013). Epigenetic effects of dietary butyrate on hepatic histone acetylation and enzymes of biotransformation in chicken. *Acta Veterinaria Hungarica*.

[152] Wippermann A., Rupp O., Brinkrolf K., Hoffrogge R., Noll T. (2017). Integrative analysis of DNA methylation and gene expression in butyrate-treated CHO cells. *Journal of Biotechnology*.

[153] Shin J. H., Xu L., Li R. W. (2014). A high-resolution whole-genome map of the distinctive epigenomic landscape induced by butyrate in bovine cells. *Animal Genetics*.

[154] Lin M. Y., De Zoete M. R., Van Putten J. P., Strijbis K. (2015). Redirection of epithelial immune responses by short-chain fatty acids through inhibition of histone Deacetylases. *Frontiers in Immunology*.

[155] Paskova L., Smesny-Trtkova K., Fialova B., Benedikova A., Langova K., Kolar Z. (2013). Different effect of sodium butyrate on cancer and normal prostate cells. *Toxicology in Vitro*.

[156] Shin H., Kim J. H., Lee Y. S., Lee Y. C. (2012). Change in gene expression profiles of secreted frizzled-related proteins (SFRPs) by sodium butyrate in gastric cancers: induction of promoter demethylation and histone modification causing inhibition of Wnt signaling. *International Journal of Oncology*.

[157] Desgagné V., Guérin R., Guay S. P. (2017). Changes in high-density lipoprotein-carried miRNA contribution to the plasmatic pool after consumption of dietary trans-fat in healthy men. *Epigenomics*.

[158] Flores-Sierra J., Arredondo-Guerrero M., Cervantes-Paz B. (2016). The trans fatty acid elaidate affects the global DNA methylation profile of cultured cells and *in vivo*. *Lipids in Health and Disease*.

[159] Thakur V. S., Deb G., Babcook M. A., Gupta S. (2014). Plant phytochemicals as epigenetic modulators: role in cancer chemoprevention. *AAPS Journal*.

[160] Amaral C. L., Milagro F. I., Curi R., Martínez J. A. (2014). DNA methylation pattern in overweight women under an energy-restricted diet supplemented with fish oil. *BioMed Research International*.

[161] Azaizeh H., Saad B., Cooper E., Said O. (2008). Traditional Arabic and Islamic Medicine (TAIM) now joins CAM, Kampo, and Ayurveda. *Evidence-Based Complementary and Alternative Medicine*.

[162] Saad B., Ramazan I. (2015). Integrating traditional Greco-Arab and Islamic herbal medicine in research and clinical practice. *Phytotherapies: Safety, Efficacy, and regulation*.

[163] Azaizeh H., Saad B., Khaleel K., Said O. (2006). The state of the art of traditional Arab herbal medicine in the Eastern region of the Mediterranean: a review. *Evidence-Based Complementary and Alternative Medicine*.

[164] Saad B., Azaizeh H., Abu Hijleh G., Said O. (2006). Safety of traditional Arab herbal medicine. *Evidence-Based Complementary and Alternative Medicine*.

